# The Impact of Endovascular Rescue Therapy on the Clinical and Radiological Outcome After Aneurysmal Subarachnoid Hemorrhage: A Safe and Effective Treatment Option for Hemodynamically Relevant Vasospasm?

**DOI:** 10.3389/fneur.2022.838456

**Published:** 2022-05-09

**Authors:** Dorothee Mielke, Katja Döring, Daniel Behme, Marios Nikos Psychogios, Veit Rohde, Vesna Malinova

**Affiliations:** ^1^Department of Neurosurgery, Georg-August-University Göttingen, Göttingen, Germany; ^2^Department of Neuroradiology, Georg-August-University Göttingen, Göttingen, Germany; ^3^Department of Neuroradiology, Otto von Guericke University, Magdeburg, Germany; ^4^Department of Neuroradiology, University Hospital Basel, Basel, Switzerland

**Keywords:** subarachnoid hemorrhage, cerebral vasospasm, endovascular therapy, outcome, endovascular treatment

## Abstract

**Objective:**

Cerebral vasospasm (CVS) represents one of the multiple contributors to delayed cerebral ischemia (DCI) in patients with aneurysmal subarachnoid hemorrhage (aSAH). Especially the management of CVS, refractory to medical treatment, is a challenging task during the acute phase after aSAH. Endovascular rescue therapies (ERT), such as medical and mechanical dilation, are possible treatment options on an individual basis. However, data about the influence on the patients' functional outcomes are limited. This study aims to assess the impact of ERT on the long-term functional outcome in aSAH-patients with refractory CVS.

**Methods:**

We performed a retrospective analysis of aSAH patients treated between 2012 and 2018. CVS was considered refractory, if it persisted despite oral/intravenous nimodipine application and induced hypertension. The decision to perform ETR was made on an individual basis, according to the detection of “tissue at risk” on computed tomography perfusion (CTP) scans and CVS on computed tomography angiography (CTA) or digital subtraction angiography (DSA). The functional outcome was assessed according to the modified Rankin scale (mRS) 3 months after the ictus, whereas an mRS ≤ 2 was considered as a good outcome.

**Results:**

A total of 268 patients were included. Out of these, 205 patients (76.5%) were treated without ERT (group 1) and 63 patients (23.5%) with ERT (group 2). In 20 patients (31.8%) balloon dilatation was performed, in 23 patients (36.5%) intra-arterial nimodipine injection alone, and in 20 patients (31.8%) both procedures were combined. Considering only the patient group with DCI, the patients who were treated with ERT had a significantly better outcome compared to the patients without ERT (Mann–Whitney test, *p* = 0.02).

**Conclusion:**

Endovascular rescue therapies resulted in a significantly better functional outcome in patients with DCI compared to the patient group treated without ETR. CTP and CTA-based identification of “tissue at risk” might be a reliable tool for patient selection for performing ERT.

## Introduction

Cerebral vasospasm (CVS) is a common finding in patients with aneurysmal subarachnoid hemorrhage (aSAH), typically occurring between day 4 and 14 after the initial bleeding, reaching a peak incidence on day 7, bearing the risk of deteriorating the outcome of affected patients (1). Although CVS is angiographically detected in up to 70% of patients with aSAH, only 30% of them develop symptoms attributable to large vessel narrowing with subsequent brain tissue hypoperfusion and ischemia, manifesting in up to 7% of the cases (2). Furthermore, delayed cerebral ischemia (DCI) can be devastating aftermath of CVS (3, 4). Several studies have proven a significant reduction of CVS using vasodilators without subsequently improving the patients' outcomes. Consequently, a paradigm shift has taken place in SAH-research from mono- to multicausal DCI-pathomechanisms (5–7). Due to increasing insights into DCI-pathogenesis, the prediction of DCI has become even more complex (8, 9). Overall, CVS represents only one of the multiple contributors to DCI. In case of hemodynamically relevant large vessel narrowing, CVS would most likely lead to significant cerebral hypoperfusion and subsequent ischemia, if CVS treatment would be held back. Currently, DCI treatment mainly consists of induced hypertension and by maintaining euvolemia (10). In case of CVS that is refractory to conservative treatment, medical and/or mechanical vasospasmolysis might be an interventional treatment option with the aim to improve cerebral perfusion with a subsequent risk reduction of persistent cerebral ischemia. Nevertheless, endovascular interventions bear a risk for complications itself, which might have a negative impact on the patient's outcome as well. Although several studies on the interventional treatment of CVS have been published, its role as rescue therapy for CVS, refractory to conservative management, has not been clearly defined up to date (11–15). A great variance exists regarding the applied criteria for the initiation of endovascular treatment, its timing, and the type of intervention as well. Hence, the formulation of clear recommendations for interventional rescue therapy in patients with CVS is still not justified based on the current literature. It is crucial that hemodynamically relevant CVS is being detected early and reliably to justify an interventional treatment. Computed tomography perfusion (CTP) has been increasingly used during the last years for the prediction of DCI and the detection of “tissue at risk” in patients with aSAH (16). However, due to the associated radiation exposure, CTP is not suitable for continuous monitoring of cerebral perfusion and is usually performed once DCI is being suspected with the aim to confirm the diagnosis and for the initiation of further treatment. It has yet to be determined whether a CTP-based imaging protocol is a reliable tool to constitute the indication for endovascular intervention as a rescue treatment for refractory CVS. This study aims to evaluate the impact of endovascular intervention as rescue therapy for severe CVS on clinical as well as the radiological outcome of aSAH-patients, with an indication for or against rescue treatment based on an elaborated imaging protocol of CTP that was consistently used at our department during the whole study period.

## Methods

### Patient Population

We performed a retrospective analysis of consecutive patients with aSAH, treated between January 2012 and December 2018. The clinical status by the time of admission was documented using the World Federation of Neurosurgical Societies (WFNS) grading system. aSAH was confirmed by computed tomography angiography (CTA) and/or digital subtraction angiography (DSA). The aneurysm was secured either by microsurgical clipping or endovascular coiling within 48 h after the initial ictus. An unenhanced computed tomography (CT) scan was done within 24 h after aneurysm treatment to exclude treatment-associated complications, such as hemorrhage and infarction. All patients were treated for at least 2 weeks in the interdisciplinary intensive care unit according to a pre-defined protocol for the acute management of patients with aSAH. Nimodipine was preventatively administered (orally or intravenously) in all patients during the first 2 weeks. A neurological examination was carried out in all awake patients at least twice a day and a neurological deterioration was defined as delayed ischemic neurological deficits (DIND), if other possible causes, such as hydrocephalus, metabolic disorders, and epileptic seizures, could be ruled out. Transcranial Doppler sonography (TCD) was performed in all patients on a daily base, given a BFV-acceleration of >120 cm/s for the middle cerebral artery considered as TCD-CVS. Arterial narrowing on CTA or DSA of at least 50% was considered angiographic CVS, whereas a vessel narrowing of >75% was defined as severe angiographic CVS. The occurrence of DIND with concurrent perfusion deficits on CTP imaging and/or a new manifestation of infarction (CT), after exclusion of treatment-associated infarctions, was defined as a DCI. The functional outcome was assessed according to the modified Rankin scale (mRS) at least 3 months after the ictus, whereat an mRS ≤ 2 was considered as a good outcome. The primary outcome parameters were DCI-associated infarction and functional outcome at 3 months.

### Imaging Protocol for the Acute Management of Patients With aSAH

In 2012, an imaging protocol was drawn up interdisciplinary (neurosurgery, neuroradiology, and intensive care medicine) at our institution to guarantee uniform management of patients with aSAH in the acute phase after the ictus. A CT combined with CTA and CTP was performed routinely in all comatose/sedated patients on day 3 and day 7 after the bleeding event. Additionally, CT, CTA, and CTP were done in cases of DIND or TCD-VSP, and in case of an increase in BFV-acceleration of >50 cm/s within 24 h. The CTP parameters were analyzed qualitatively and quantitatively using cutoff values (time to drain > 4.93 s, cerebral blood flow <53.93 ml/100 ml/min, mean transit time > 4.25, time to start > 9.28 s, cerebral blood volume <3.14 ml/100 ml), which were previously published elsewhere (16).

### Indication Criteria to Perform Endovascular Intervention as Rescue Therapy for Refractory CVS

In patients with CVS, diagnosed by TCD or CTA/DSA, medical treatment consisting of induced hypertension was initiated with a target systolic arterial pressure of 160–180 mmHg. If large vessel CVS (>50% vessel lumen narrowing) was detected and associated with DIND and/or perfusion deficits on CTP that is refractory to medical treatment, the patient was selected for endovascular intervention. The indication to perform ERT was based on multiple paramaters (severe angiographic CVS, clinical deterioration, and perfusion deficits confirmed by CTP). In comatose or sedated patients ERT was indicated based on the presence of severe angiographic CVS as well as perfusion deficits confirmed by CTP. The presence of TCD-VSP or angiographic CVS alone without clinical deterioration or perfusion deficits was not considered an indication for ERT.

**Figure 1:**
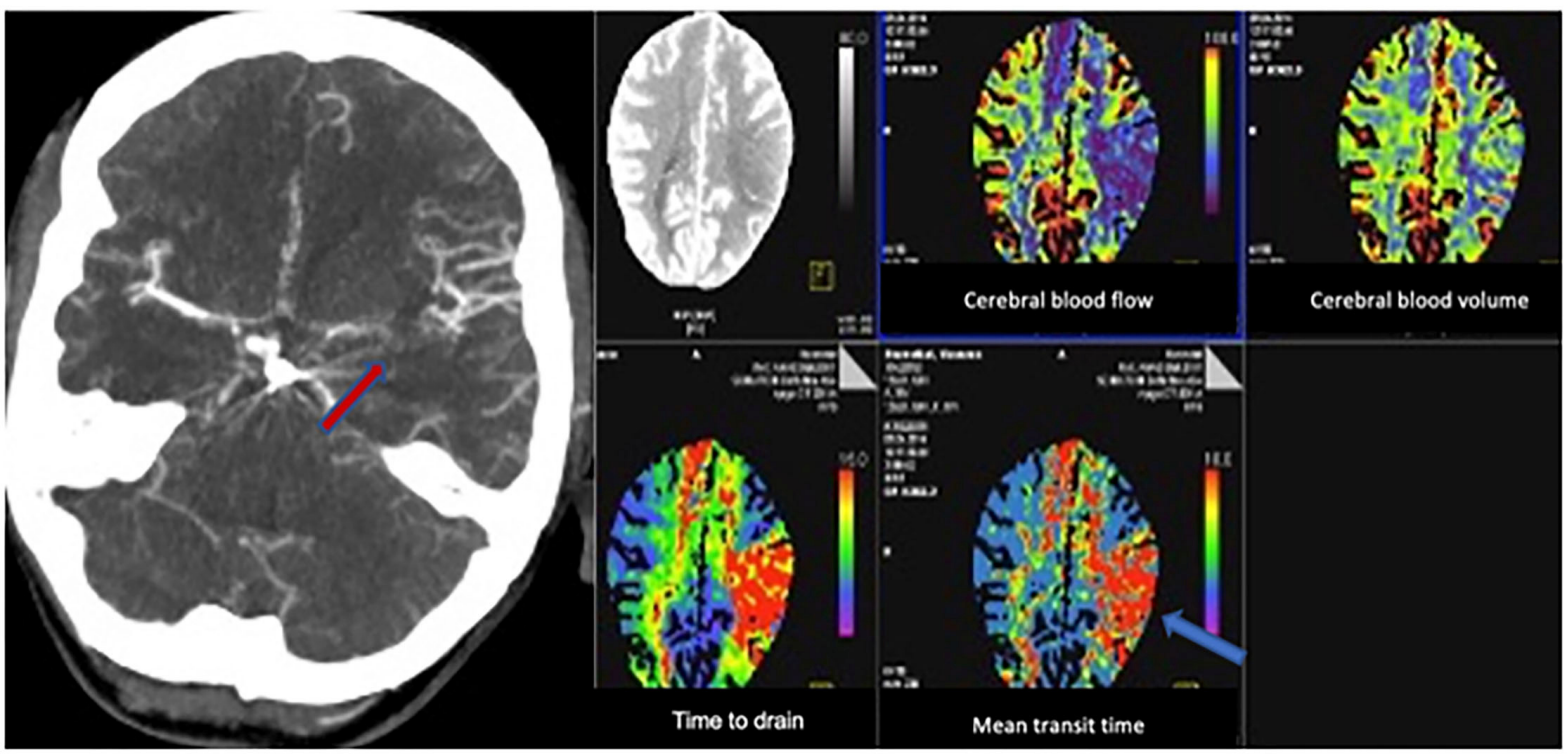
Example of a patient with an indication for ERT with severe vasospasm on the CTA (red arrow, left) with perfusion deficits in the middle cerebral artery territory on the CTP (blue arrow, right).

Figure 1 shows an example of a patient with an indication for ERT due to hemodynamically relevant angiographic vasospasm associated with manifested perfusion deficits on the CTP. The endovascular interventions included medical as well as mechanical vasospasmolysis. A medical vasospasmolysis was performed by intra-arterial application of nimodipine, whereat a mechanical vasospasmolysis was done by intra-arterial balloon angioplasty. The decision concerning the choice of ERT was made dependent on the localization of CVS. Mechanical vasospasmsmolysis was performed only if CVS was located within the internal carotid artery, the A1 segment of the anterior cerebral artery or the M1 segment of the middle cerebral artery. Multiple vessels were treated within one session, if more vessels were simultaneously affected by severe refractory vasospasm. Medical vasospasmolysis was performed in patients with vasospasm located in the distal segments of the affected cerebral arteries. In patients with simultaneous detection of proximal and distal CVS, both procedures were combined.

### Statistical Analysis

The statistical analyses were performed by means of the GraphPad Prism software (Version 8, GraphPad Software, San Diego, CA, USA). For the presentation of baseline data, descriptive statistics and frequency distribution analysis were done. Fisher's Exact test and *t*-test were performed to evaluate differences in the baseline characteristics between the patient groups. Multivariate logistic regression analysis was performed to identify independent predictors. Differences between the two groups concerning the incidence of DCI-associated infarction and the clinical outcome at 3 months follow up Fisher's Exact test was applied. One-way ANOVA analysis was performed to assess differences between more than two patient groups. For all analyzed parameters complete data were available.

## Results

### Patient Population

A total of 268 consecutive patients with aSAH, treated at our department, between January 2012 and December 2018 were included into the analysis of this study. The mean age was 55.4 (SD 14.12) ranging from 23 to 90 years. Out of these, 94 (35.1%) patients were men, and 174 (64.9%) patients were women. A good WFNS grade (I–III) was found in 181 (58.2%) patients. Most patients (243; 90.7%) had a high Fisher grade (3, 4). An overview of the baseline patients' characteristics is provided in Table 1. Endovascular rescue therapy was performed in 63 (23.5%) of all patients. We compared the baseline characteristics between the patients with and without ERT and found no statistically significant differences (Table 1).

**Table 1:** Baseline characteristics of the study population.

**Parameters**	**All**	**Without ERT**	**With ERT**	***P*-value**
Mean age (SD) in years	55.4 (14.1)	56.3 (14.6)	52.8 (12.3)	0.37
**Sex**				
Male (%)	94 (35.1%)	70 (34%)	24 (38%)	0.65
Female (%)	174 (64.9%)	135 (66%)	39 (62%)
**WFNS**				
Grade I-III	156 (58.2%)	121 (59%)	35 (55.6%)	0.66
Grade IV-V	112 (41.8%)	84 (41%)	28 (44.4%)
**Fisher**				
Grade 1-2	25 (9.3%)	23 (11.2%)	2 (3.2%)	0.07
Grade 3-4	243 (90.7%)	182 (88.8%)	61 (96.8%)
**Aneurysm location**				
Anterior circulation	229 (85.5%)	175 (85.4%)	54 (85.7%)	0.99
incl. PCommA
Posterior circulation	39 (14.6%)	30 (14.6%)	9 (14.3%)
**Aneurysm treatment**				
Clipping	144 (53.7%)	108 (52.7%)	36 (57.1%)	0.56
Coiling	124 (46.3%)	97 (47.3%)	27 (42.9%)

*PCommA, posterior communicating artery*.

### Incidence of TCD-Vasospasm, DIND, DCI, and DCI-Associated Infarction

Transcranial Doppler sonography-vasospasm was detected in 118 patients (44%), 60 patients (22.4%) developed a DIND, and DCI was diagnosed in 86 patients (32.1%). DCI-associated infarction was detected in 51 patients (19%). As expected, we found a higher incidence of TCD-VSP (79.4 vs. 33.3%, *p* < 0.0001) and DIND (39.7 vs. 17.1%, *p* < 0.0001) in the patient group that received ERT compared to the patient group without ERT.

### Endovascular Rescue Therapy Data

**Figure 2:**
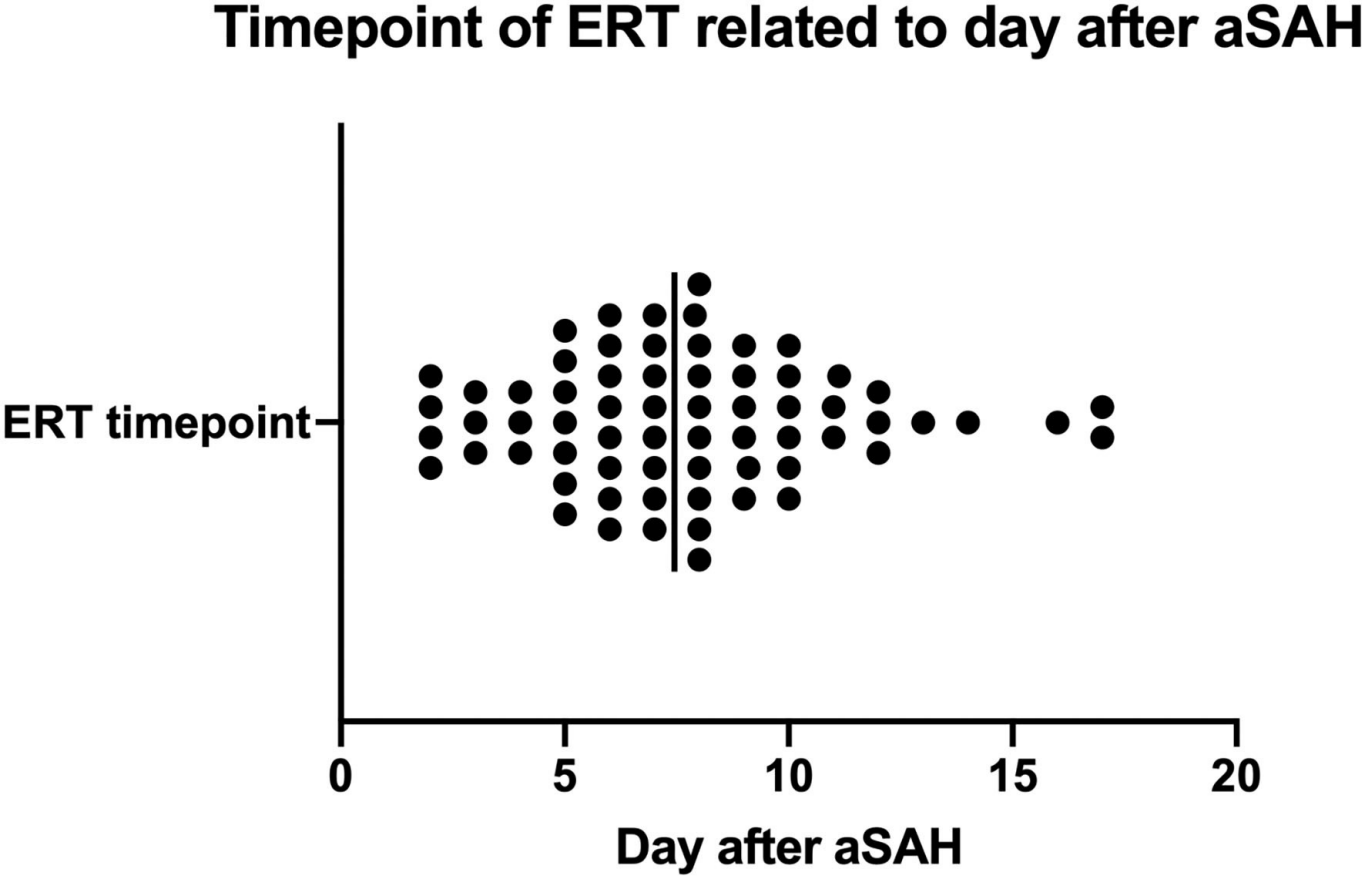
Timepoint at which ERTs were performed in relation to the timepoint of the bleeding event (the day after aSAH diagnosis).

**Figure 3:**
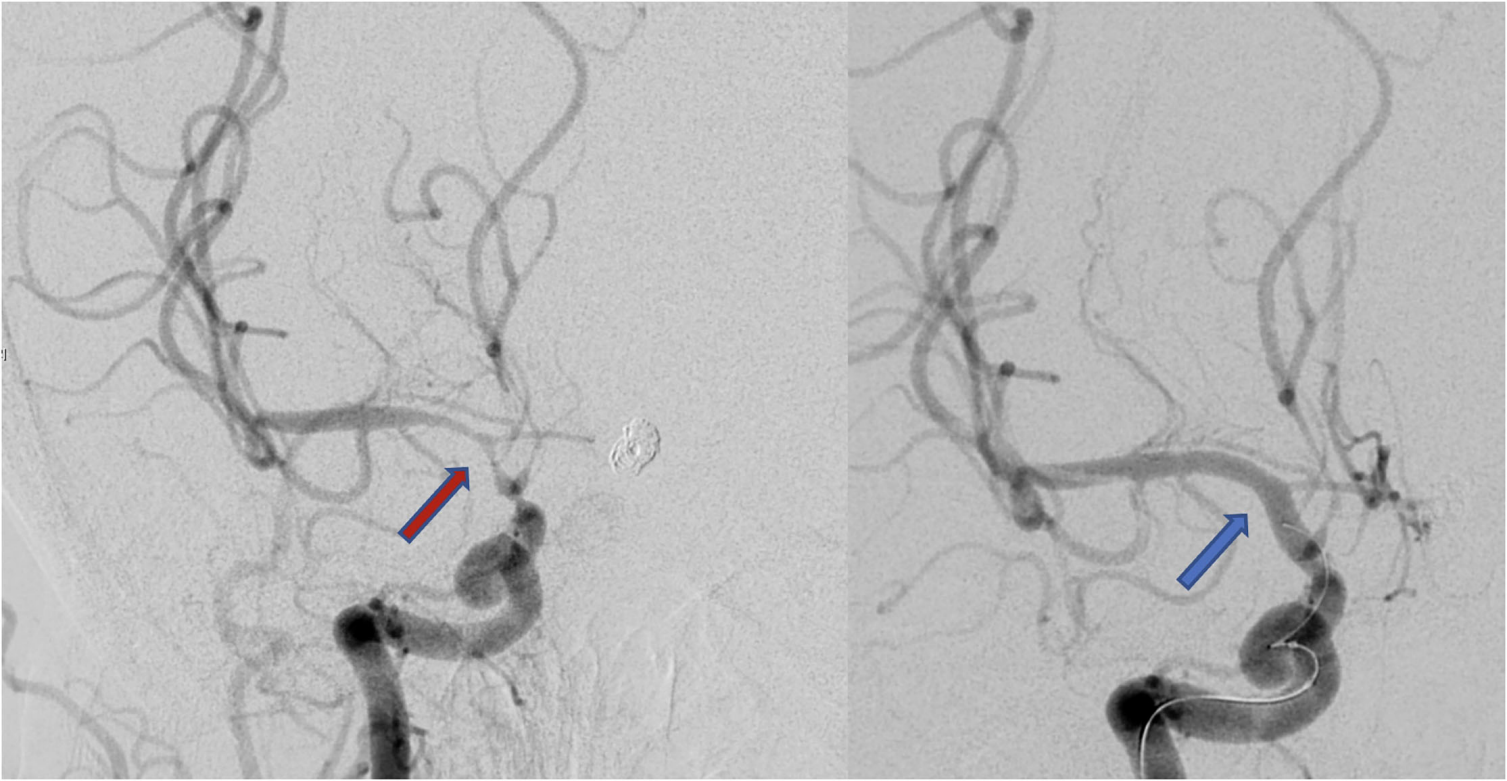
Conventional angiography showing severe vasospasm of the right internal carotic artery, middle cerebral artery, and anterior cerebral artery before (red arrow, left) and after (blue arrow, right) the intervention.

Endovascular rescue therapy was performed in 63 (73%) of the 86 patients with DCI, and 23 patients with DCI received only induced hypertension without ERT. In 20 patients (31.8%) balloon dilatation was performed, in 23 patients (36.5%) intra-arterial nimodipine injection alone, and in 20 patients (31.8%) both procedures were combined. The mean overall nimodipine dose per ERT was 4.09 mg (SD 3.05 mg), ranging from 1 to 14 mg. Considering the applied balloons, in 23 patients a compliant balloon was used, in 12 patients a semi-compliant balloon, and in the rest of 8 patients the model of the used balloon was not documented. Up to three ERTs were performed per patient: 47 patients (74.6%) received one ERT, 12 patients (19.1%) had 2 ERTs, and in 4 patients (6.4%) ERT was performed thric. The mean radiation exposure per ERT procedure was 33.5 mSv (SD 25.7 mSv), ranging from 10.3 to 218.6 mSv. ERT was performed on average on day 7 (mean 7.7 days, SD 3.4, range 2–17 days after aSAH) (Figure 2). All 63 patients with ERT had angiographic vasospasm [71.4% moderate (vessel lumen reduction of 50–75%) and 28.6% severe (vessel lumen reduction of >75%)]. In 33 patients (52.4%) perfusion deficits were diagnosed (in 78.8% territorial deficits and in 21.2% diffuse unspecific perfusion deficits). By the time of ERT, 12 out of 63 patients were awake and developed a DIND. In 10 out of these 12 patients the neurological deficits completely resolved after the ERT intervention. Two patients suffered from permanent deficits. TCD-VSP was present in 79.4% of the patients with ERT. We found a significant reduction in blood flow velocities measured by TCD before (mean BFV 174.2 cm/s SD 30.02, range 130–256 cm/s) and after (mean 104.9 cm/s SD 27.6, range 54–160 cm/s) the intervention (*t*-test, *p* < 0.0001). The angiographic vasospasm resolved in all patients after the intervention (Figure 3).

### Clinical and Radiological Outcome Parameter

**Figure 4:**
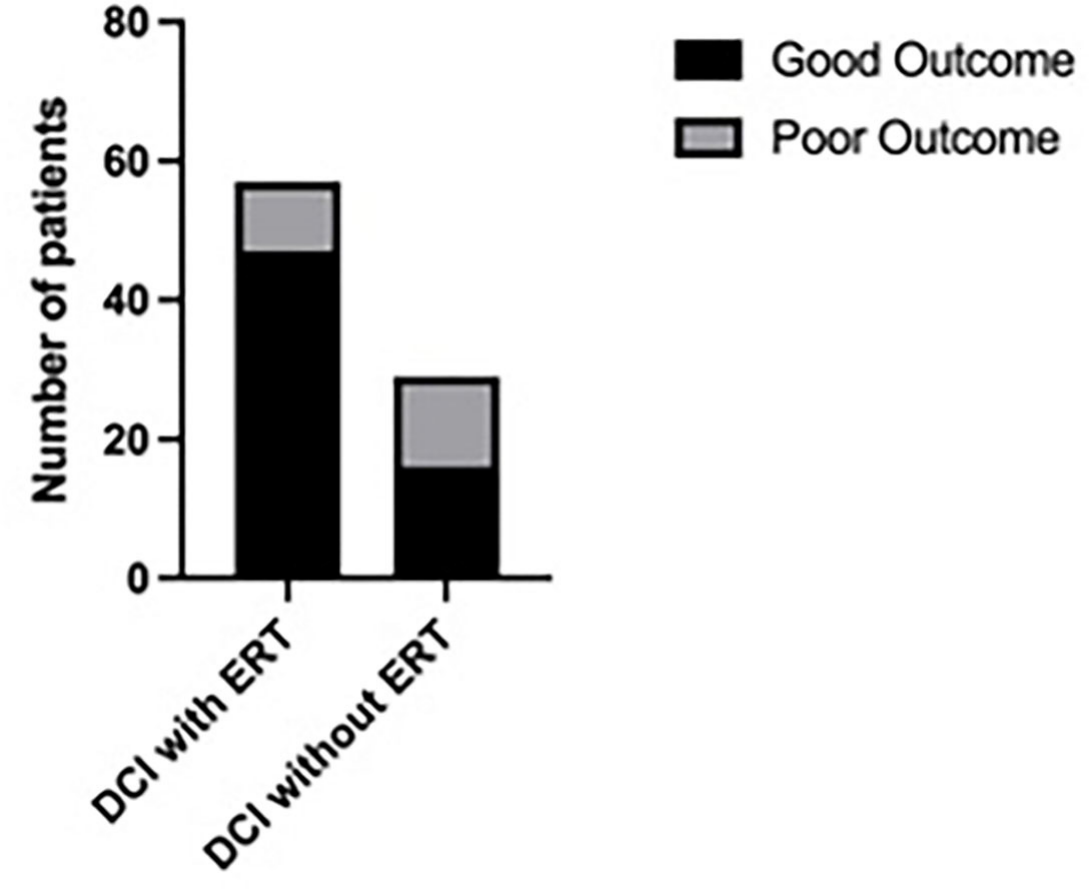
Comparison of functional outcome of patients with DCI receiving ERT with the patient group with DCI without ERT, showing a significantly higher percentage of patients with good outcome (mRS ≤ 2) in the patient group with DCI and ERT compared to the patient group with DCI without ERT (Fisher's Exact test, *p* = 0.01).

After 3 months, a good clinical outcome (mRS ≤ 2) was documented in 202 patients (75.4%). In the patient group that underwent ERT an mRS ≤ 2 was found in 82.5% (52/63) and in 73.2% (150/205) in the patient group without ERT, respectively. In the multivariate logistic regression analysis, independent outcome predictors were age (*p* = 0.001), WFNS grade (*p* = 0.003), and DCI-associated infarction (*p* = 0.001), Table 2. Considering only the patient group with DCI, the patients who received ERT had a significantly better outcome compared to the patients without ERT (Fisher's Exact test, *p* = 0.01, Figure 4). The incidence of DCI-associated infarction was lower in the patient group with DCI and ERT (32.7%) compared to the group with DCI and without ERT (46.7%), but the difference was not significant (Fisher's Exact test, *p* = 0.48). In the multivariate logistic regression analysis, independent predictors of DCI-associated infarction were WFNS grade (*p* = 0.0005), DIND (*p* < 0.0001), and DCI (*p* = 0.006), Table 3.

**Table 2:** Multivariate logistic regression analysis for identification of predictors of good clinical outcome (mRS ≤ 2).

**Variables**	**OR**	**95% Confidence interval**	***P*-value**
Age	0.961	0.938-0.984	0.001
Lower WFNS-grade (I-III)	2.630	1.397-5.029	0.003
Lower Fisher-grade (1,2)	1.982	0.601-9.039	0.30
Aneurysm location (anterior vs. posterior circulation	0.908	0.364-2.144	0.83
Aneurysm treatment (clipping vs. coiling)	1.004	0.520-1.927	0.99
Presence of TCD-vasospasm	1.254	0.609-2.621	0.54
Presence of DIND	1.353	0.583-3.338	0.49
Received ERT	2.494	0.921-7.096	0.07
Presence of DCI-associated infarction	0.224	0.089-0.533	0.001

*WFNS, World Federation of Neurosurgical Societies; TCD, transcranial Doppler sonography; DCI, delayed cerebral ischemia; DIND, delayed ischemic neurological deficits; ERT, endovascular rescue therapy; DCI, delayed cerebral ischemia*.

**Table 3:** Multivariate logistic regression analysis for identification of predictors of DCI-associated infarction.

		**95%**
**Variables**	**OR**	**Confidence interval**	***P*-value**
Presence of	1.381	0.576-3.315	0.46
TCD-vasospasm
Presence of DCI	3.126	1.387-7.217	0.006
Presence of DIND	10.830	4.979-24.860	<0.0001
Lower WFNS-grade (I-III)	0.238	0.102-0.520	0.0005
Lower Fisher-grade (1,2)	0.732	0.098-3.388	0.71
Age	1.021	0.992-1.052	0.16

*TCD, transcranial Doppler sonography; DCI, delayed cerebral ischemia; DIND, delayed ischemic neurological deficits; WFNS, World Federation of Neurosurgical Societies*.

## Discussion

Fortunately, the introduction of improved management strategies for the treatment of aSAH reduced the mortality rate of patients with SAH during the last decades (17, 18). It remains unquestionable that DCI is a major cause of morbidity and mortality after SAH and that available treatment options should be exploited to counteract DCI (2). Up to date, sparse evidence exists, and no commonly accepted treatment guidelines are available to counteract refractory CVS (19). For many years, one mainstay has been Triple-H-therapy (20). Through ongoing research, Triple-H-therapy consisting of hypertension, hemodilution, and hypervolemia has been downgraded to Mono-H-Therapy, with the remaining pier hypertension. Surprisingly, induced hypertension has been used for decades to promote CBF under vasospastic conditions, although no randomized clinical trial existed to support its use (21). Despite several limitations, the use of this last remaining anchor has even been questioned after the publication of the HIMALAIA-trial, which could not demonstrate an improvement of cerebral perfusion by induced hypertension (22). Concerning this trial, it has to be mentioned, that only 41 patients have been randomized by the time of termination and the results have to be interpreted very carefully. Consequently, it is inevitable to raise the focus on other available treatment options, among which is ERT (19). However, evidence in this context is scarce (23). Only retrospective case series focusing on the treatment of CVS using intra-arterial chemical agents for spasmolysis, balloon angioplasty, and more recently stent-retrievers have been published.

Furthermore, no clear criteria for indicating ERT has been defined so far. A major limitation in previously published studies, as well as in our study is also the lack of a control group with a randomization of patients with refractory CVS for ERT or against ERT, which represents an ethical issue. In our series, a pre-defined protocol based on CTP imaging was applied as an indicator for hemodynamically relevant CVS suitable for ERT, which separates our study from previously published studies addressing this question. The CTP-protocol was adapted for the prediction of DCI and detection of “tissue at risk” in awake as well as in sedated patients. While CTP was triggered by clinical deterioration (DIND) or TCD-VSP in awake patients, routine CTP was carried out at two pre-defined timepoints (day 3 after ictus before the beginning of the “VSP phase” and day 7 as the peak of the “VSP phase”) additionally to TCD-VSP as a trigger. Bele et al. presented an alternative approach based on invasive multimodal monitoring for indicating continuous intraarterial nimodipine application to treat refractory vasospasm in comatose patients using bilateral measurement of partial pressure of tissue oxygenation (PbtO2) and cerebral blood flow (CBF) monitoring (Bowman Perfusion Monitor) (24). DSA was performed if a PbtO_2_ > 15 mmHg and a CBF > 20 ml/100 g/min could not be achieved, whereas continuous intraarterial nimodipine application was indicated in case of severe CVS. The advantage of CTP over local invasive monitoring is the coverage of whole-brain perfusion and its non-invasiveness. A disadvantage of CTP is the associated radiation exposure. Another disadvantage is the static nature of imaging not allowing continuous monitoring.

The key finding in our large retrospective case series was that regardless of the applied method, the patients' outcome was significantly better when ERT was performed in the presence of CVS. Due to the retrospective data analysis, we can not reconstruct the exact reasons for not indicating ERT in 23 patients, which is a limitation of the presented study. However, it seems reasonable that patients selected for ERT must have been regarded as more severely affected with a higher risk for developing infarction in comparison to patients in whom no indication for ERT was seen. Nevertheless, they still have experienced better outcomes, which would support the beneficial effect of ERT in this patient population.

### Chemical Spasmolysis

With regards to the presented data, 36.5% of the patient population received solely an intra-arterial nimodipine application. In all cases, angiographic vasospasm resolved completely. Unfortunately, we are unable to provide information on duration of spasmolysis. In all cases, intraarterial nimodipine was applied with a mean dosage of approximately 4 mg. In general, papaverine and nimodipine have been mostly used for chemical spasmolysis. Milrinone, nicardipine, verapamil, and fasudil are further reported agents in the literature (25, 26). In recent years, the use of papaverine has declined due to detected paradoxical vasospasm, systemic hypotension, and drug toxicity. Verapamil, which also has a short half-life and selectively reacts with myocardium, may be better suited for treating coronary vasospasm (27). Not to be concealed is the fact that no standard dosing regimen exists for the administration of intra-arterial agents, challenging its' utilization in clinical practice even more (19). Some prefer a long, slow administration time, while others administer the medication as a bolus (20). Within the presented study, patients received a mean nimodipine dose of 4.1 mg per ERT, administered as a bolus. Although nimodipine and nicardipine were found to have a longer duration of action than verapamil in terms of vasodilation, their effects were not sustained beyond 2 h after intraarterial infusion in a rabbit animal model. Again, these results are in line with the findings by Vajkoczy and colleagues, as discussed before (27, 28). Having in mind the lacking long-term efficacy, selective continuous intra-arterial infusion, as recently described in the case of nimodipine, seems to be an alternative (29). Furthermore, the hypotensive effect of spasmolytic agents should not be underestimated. The hypotensive effect of intra-arterial nicardipine was documented in a high proportion of patients, with the need to increase or initiate vasopressor therapy. Besides, only the nicardipine group showed a significant 3-h period of lowered blood pressure within an experimental setting (27). Given the lack of controlled studies comparing different agents, the recommendation for one or another chemical vasodilators would require prospective randomized studies (30). There was no complication related to ERT-induced hypotension within this study.

### Transluminal Balloon Angioplasty

Most patients included in our study underwent TBA or received a combined treatment of TBA and chemical spasmolysis. TBA was first described in 1984 for the treatment of severe CVS (31). Initially, TBA was generally not considered to be safe beyond the carotid or M1 segment (32). Besides, the use of non-compliant rather than compliant balloons has been suggested to prevent overdilation and injury of the artery (33). A couple of years ago, it was assumed that this proposition may change with the introduction of newer balloon catheters that are safer in more distal segments (20, 34, 35). In the meantime, non-compliant balloons were reported to be associated with a higher rate of recurrent vasospasm or failure of angioplasty or dissection because of difficulties in choosing the right diameter of the balloon as well as challenging navigation of more rigid balloon catheters (36). Nowadays, several studies have proven the use of compliant or even hyper-compliant balloons for TBA (11, 35). As expected, TBA was recently performed in terms of distal TBA, up to the second segment of cerebral arteries. In a recent study, proximal TBA and distal chemical angioplasty was compared to simultaneous proximal and distal TBA. A better efficacy, while maintaining safety, was described in the setting of sole TBA. Besides a reduction in the incidence of DCI, the authors were not able to show a significant impact on the clinical outcome, again supporting the theory of multicausal pathogenesis of DCI (36). In this study, several techniques of TBA have been implemented, thus making it difficult to provide recommendations for one specific technique of TBA. Recently, the use of stent-retrievers, developed for thrombectomy in stroke patients, has been described as a technically feasible method for the treatment of refractory CVS (37). Unfortunately, the number of patients in the study is too small to prove statistical significance.

### Timing of Endovascular Rescue Therapy

In the clinical setting, it appears that TBA is most effective when applied early after the diagnosis of vasospasm (38). This assumption is being supported by findings in experimental settings, where TBA, applied to vessels before the onset of vasospasm, was found to completely prevent vasospasm (39). Transferred to clinical practice it should be considered that prophylactic treatment can be associated with potential risks and up to date no significant effects on DCI and outcome have been proven (35). On the other hand, evidence exists that the need for emergency intra-arterial treatment is being reduced by prophylactic treatment (32). Through utilizing frequent and early ERT, the risk of DCI can be reduced as well as an improvement in the patients' functional outcome can be achieved (40). These findings are in conjunction with a recently published prospective analysis (41). Herein, timely ERT provided a relatively safe and effective treatment option in highly selected patients undergoing multimodality monitoring. Continuous treatment, in particular, may be suitable to surpass sustained DCI, as it was associated with a low rate of DCI-related infarction and a relatively high percentage of good outcome (41). We implemented a standard operating procedure (SOP), including CTA and CTP, on day 3 and 7 after the bleeding event, to monitor closely patients at risk for CVS. In a recent study in which we compared outcome data before and after the establishment of the SOP, we could show that we achieved a better outcome after applying the SOP, and that we indicated more often ERT. This allows Us to draw the conclusion that we identified tissue at risk earlier and more frequently, supporting the concept, that timing plays a crucial role for containing satisfying results with ERT.

## Conclusion

Patients with DCI, defined as cerebral vasospasm associated with DIND or perfusion deficits on CTP that is refractory to medical treatment, who received ERT have experienced a significantly better functional outcome in our study compared to DCI patients with refractory vasospasm receiving maximal conservative treatment without ERT. Since ERT is associated with radiation exposure and other procedure-related complications, elaborated selection criteria are essential for patient selection. In this context, a CTP and CTA-based identification of “tissue at risk,” as applied in our study, may serve as a reliable tool for patient selection and timing of ERT, which merits an evaluation in a prospective setting to facilitate a comprehensible decision-making process for indicating ERT.

## Data Availability Statement

The original contributions presented in the study are included in the article/supplementary materials, further inquiries can be directed to the corresponding author/s.

## Ethics Statement

This study was conducted according to the principles of the Helsinki Declaration. The study (Number: 16/9/20) was approved by the Local Ethics Committee of the University Medicine Göttingen. A patient's consent for treatment was obtained according to the individual institutional guidelines. Due to a retrospective analysis of the data for this study additional informed consent was deemed unnecessary.

## Author Contributions

DM contributed to data interpretation and manuscript writing. KD was involved in data acquisition and data analysis. DB and MP contributed to neuroradiological data analysis. VR contributed to data interpretation and supervision. VM contributed to data analysis and interpretation, manuscript writing, and supervision. All authors read and critically reviewed the final version of the manuscript.

## Conflict of Interest

The authors declare that the research was conducted in the absence of any commercial or financial relationships that could be construed as a potential conflict of interest.

## Publisher's Note

All claims expressed in this article are solely those of the authors and do not necessarily represent those of their affiliated organizations, or those of the publisher, the editors and the reviewers. Any product that may be evaluated in this article, or claim that may be made by its manufacturer, is not guaranteed or endorsed by the publisher.
